# Robust water repellent ZnO nanorod array by Swift Heavy Ion Irradiation: Effect of Electronic Excitation Induced Local Chemical State Modification

**DOI:** 10.1038/s41598-017-03313-8

**Published:** 2017-06-12

**Authors:** Kugalur Shanmugam Ranjith, Lalitha Raveendran Nivedita, Kandasami Asokan, Satheesh Krishnamurthy, Ramanathaswamy Pandian, Mohammed Kamruddin, Devesh Kumar Avasthi, Ramasamy Thangavelu Rajendra Kumar

**Affiliations:** 1 Advanced Materials and Devices Laboratory, Department of Physics, Bharathiar University, Coimbatore, 641 046 India; 2 Materials Science Division, Inter University Accelerator Centre, New Delhi, 110 067 India; 3 School of Engineering and Innovation, The Open University, Milton Keynes, MK76AA UK; 4 Surface and Nanoscience Division, Materials Science Group, Indira Gandhi Centre for Atomic Research (IGCAR), Kalpakkam, 603 102 India; 5 Amity Institute of Nanotechnology, Noida, 201313 India; 6 Department of Nanoscience and Technology, Bharathiar University, Coimbatore, 641 046 India

## Abstract

Tailoring the surface properties by varying the chemistry and roughness could be of interest for self-cleaning applications. We demonstrate the transformation of hydrophobic ZnO Nano rod (NR) array into superhydrophobic nature by changing the local chemical state and without altering the surface roughness by swift heavy ion (SHI) irradiation. The aligned ZnO NR arrays were irradiated using 150 MeV Ag ions with different fluences from 5E10 to 3E12 ions/cm^2^. The observed static water contact angles of ZnO NRs samples were 103° ± 3°, 152° ± 4°,161° ± 3°, 164° ± 2°, 167° ± 2°,154 ± 3° and 151° ± 2° for the pristine, ion fluencies of 1E11, 3E11, 5E11, 7E11, 1E12 and 3E12 ions cm^−2^, respectively. The change in local surface chemistry via formation of surface oxygen related defects due to electronic excitations induced by ion irradiation determine the water dewetting properties. It is found that surface oxygen related defects could be tuned by varying the fluence of the SHIs. Durability tests show that the SHI induced surface oxygen-deficient ZnO NRs have the stable superhydrophobic behavior for more than a year.

## Introduction

Functional materials with artificial intelligent surface with tuned and robust wetting properties such as superhydrophobicity/hydrophilicity, have been an emerging field with enormous application potential in automobile industries, display panels, medical devices, sensing platforms and other optical equipments^1–4^. The wetting properties of the surface could be altered by varying the surface roughness and surface energy by chemical functionalisation^5–7^. To date, water repellent surfaces are engineered with chemical functionalising to reduce the surface energy. The change in wettability of the surface by adding another chemical is not desirable in most of cases especially in bio-interaction platforms as the interaction of biomolecules and surface functionalised chemical groups could create additional complexity^8–11^. Therefore, methods that could alter the surface energy by inducing intrinsic local surface alterations are interesting. Among the metal oxide family, surface wetting (hydrophilic or superhydrophobic) controlled ZnO, play a crucial role in nanoscale chemical sensing, biosensing, biomedical and microfluidic applications due to its stable and tuneable chemical and physical properties^12–14^. Previous investigations reveal that under UV radiation ZnO based platforms turn hydrophilic and improved the adsorption of proteins^15, 16^. Modifying the wetting nature of ZnO nanorods (NRs) can lead to more surface interactions and, hence increased sensitivity of the sensor platform. But fabricating superhydrophobic and superhydrophilic ZnO NR based platforms without any chemical coating is essential for better performance of smart nanodevices. In this work, we demonstrate the wetting properties of ZnO NRs array that could be tailored by varying the surface energy by ion beam induced intrinsic local surface alterations.

Surface modifications such as (i) surface roughness and (ii) surface energy variation through ion beam irradiation without any external chemical functionality are potentially important in the field of materials science. The modification of material properties depends on the ion beam parameters such as mass of the ion, energy of the ion and fluence. Low energy ions (keV/amu regime) losses energy by elastic collisions with nuclei of the target material. The elastic collisions cause surface diffusion of target nuclei and influence the surface morphology resulting in formation of ripple like surface structures or increase the surface roughness of the materials^17^. On the other hand, in high energy range (MeV/amu regime), ions interact with the atomic electrons of the target material. The high energy heavy ions having energies in regime of ~MeV/u or higher are referred as swift heavy ions (SHI). The SHI transfer energy by inelastic collisions with target atomic electrons resulting in excitation of the electrons and ionization of the target atoms. The SHI generates highly energetic electron gas around their irradiation tracks. These highly energetic electrons are capable of exciting the nearby atoms and thus locally alter the chemical state of the target material.

In this work, we demonstrate the wetting properties of ZnO nanorods (NRs) array that could be tailored by varying the surface energy by ion beam induced intrinsic local surface alterations. So far, ion irradiations on 1D ZnO nanostructures were focused towards the study of change in structural, optical and electrical properties^15, 16, 18–20^. Tashlykova *et al*., reported that irradiation of Xe^+^ ion on graphite surface cause variation of surface roughness which changes its wettable properties^21^. Mostly ion irradiated polymers show change in surface roughness which resulted in modification of their wetting properties^22–28^. Tailoring the nanostructural surface by the SHI irradiation would be interesting to understand insight on electron induced local chemical modification in order to improvise the surface/interface interactions.

In the present study, ZnO NRs were synthesized and their self-affine defect states were investigated via 150 MeV Ag^11+^ ion irradiations at normal incidence. The experimental results show that ZnO NRs with oxygen- deficiency display nearly superhydrophobic wetting behavior. The role of self-affined defect states, surface modifications and wettability changes induced by ion irradiation were studied on ZnO NR arrays. The results presented here along with the earlier findings reveal an important method to generate different types of defect centres in ZnO material which will effectively contribute to the wettability variation on the surface. Understanding the nature and role of such defects in ZnO will be helpful for functional tailoring of its optical and electronic properties. The proposed water wetting mechanism over a 1D nanostructural platform with the presence of defect sites will promote its functional applications. In addition, this study reports that ion irradiation can be used to produce super hydrophobic platform of ZnO NR arrays depending on the electronic excitation induced by ion beam irradiation.

## Results

### Morphological Studies

**Figure 1:**
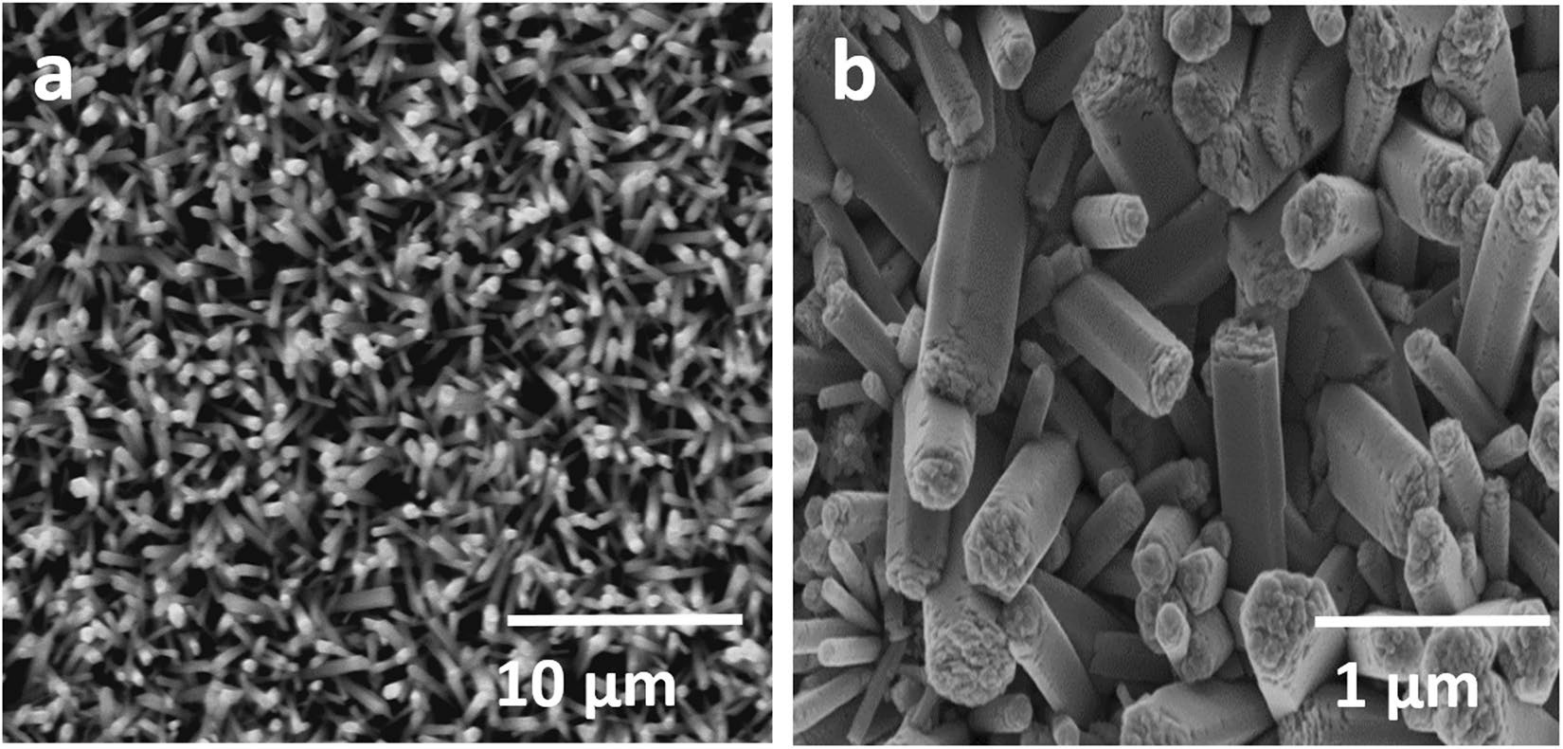
SEM top-view of solution grown ZnO NR arrays (**a**) at low magnification and (**b**) at high magnification.

Figure 1 shows the low (1a) and high (1b) magnification of the top view images of ZnO NRs grown on the dip seeded substrate. The grown NRs were having a length of ~1 μm and controlled diameter of ~100–350 nm.

### Wetting properties

**Figure 2:**
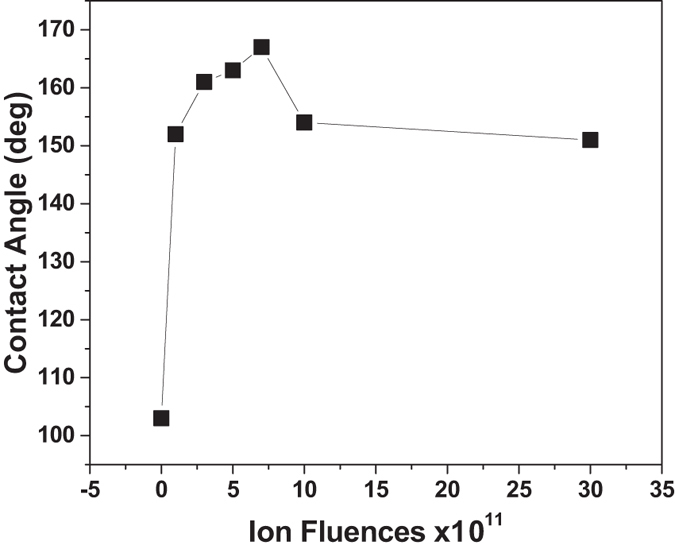
Wettability of the ZnO NR arrays irradiated under different fluences of 0, 1E11, 3E11, 5E11, 7E11, 1E12, 3E12 ions cm^−2^ with 150MeV Ag^11+^ ions.

ZnO NR arrays have been irradiated with 150 MeV Ag^11+^ ion beam with fluences of 1E11, 3E11, 5E11, 7E11, 1E12 and 3E12 ions cm^−2^. For studying the change in wettability as a function of ion beam fluences, the surface water contact angle (CA) and contact angle hysteresis (CAH) of the pristine and ion irradiated ZnO NR arrays were investigated. The wetting properties of the ion beam irradiated samples were studied by measuring the contact angle to water. Figure 2 shows water CA of the pristine and Ag ion irradiated samples. Compared with the wettability of the pristine ZnO NR arrays (CA=103° ± 3°; CAH=40° ± 3°), the ion irradiated ZnO NR arrays had enhanced CA with respect to the different irradiation fluences. On bombarding ZnO NR arrays with Ag ions of fluence 1E11 ions cm^−2^, the hydrophobic surface is changed to superhydrophobic surface with a CA and CAH of 152° ± 4° and 20° ± 2°. The CA and CAH of ZnO NR samples irradiated at fluences of 3E11, 5E11, 7E11, 1E12, 3E12 ions cm^−2^ are 161° ± 3° and 20° ± 2°; 164° ± 2° and 10° ± 2°; 167° ± 2° and 3° ± 1°; 154° ± 2° and 15° ± 2°; 151° ± 2° and 15° ± 1° respectively. Upon increasing the ion fluences, the CA is found to increase up to 7E11 ions cm^−2^. With the ion fluence of 1E12 ions cm^−2^ CA starts decreasing to 154° ± 3° but retains its superhydrophobicity. On further increasing the ion fluence as 3E12 ions/cm^2^, the CA decreases to 151° ± 2°. It is noteworthy that ZnO NRs irradiated at ion fluence as 7E11 ions cm^−2^ show higher CA (167° ± 2°) and lower CAH (3° ± 1°) indicating extreme superhydrophobic nature.

### Microstructural studies

**Figure 3:**
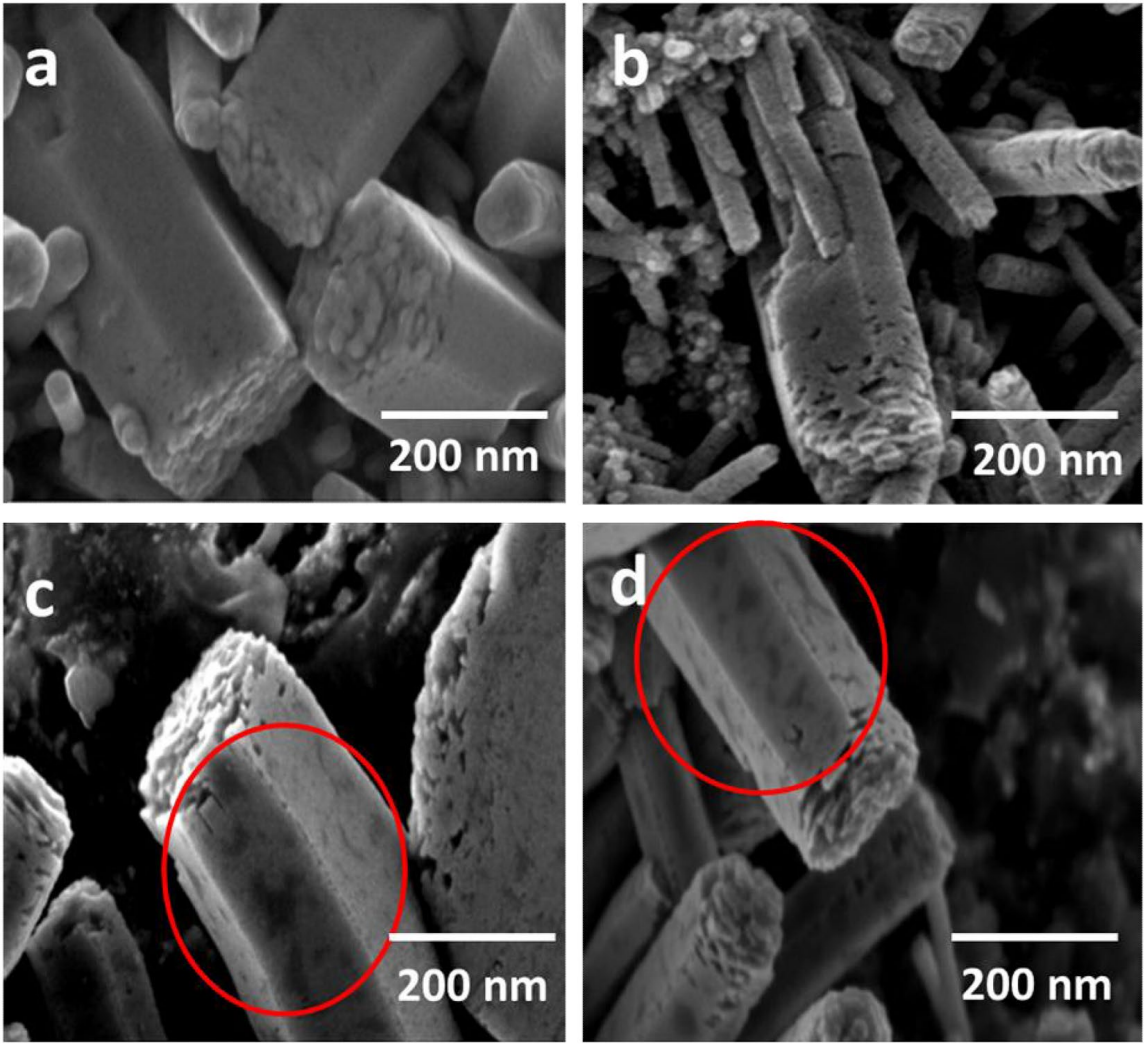
SEM images of the ZnO NRs irradiated under different fluences with 150MeV Ag^11+^ ions (**a**) pristine, (**b**) 3E11, (**c**) 7E11 and (**d**) 3E12 ions/cm^2^.The rings in the images indicate randomly distributed darker patches on the surface of the NRs.

The surface morphologies of the ZnO NRs after Ag ion irradiation are shown in the Fig. 3(a–d) i.e., pristine, 3E11, 7E11, and 3E12 ions/cm^2^ respectively. The SEM images of the ion irradiated samples show randomly distributed darker patches (as indicated in rings in the images) on the surface. These do not show any regular or periodic defect on the surface. The irradiation at different fluences brings about the change on the surface of the NR arrays depending on the induced diffusion. However, the morphology as well as size of these structures is not affected significantly by the SHI irradiations.

**Figure 4:**
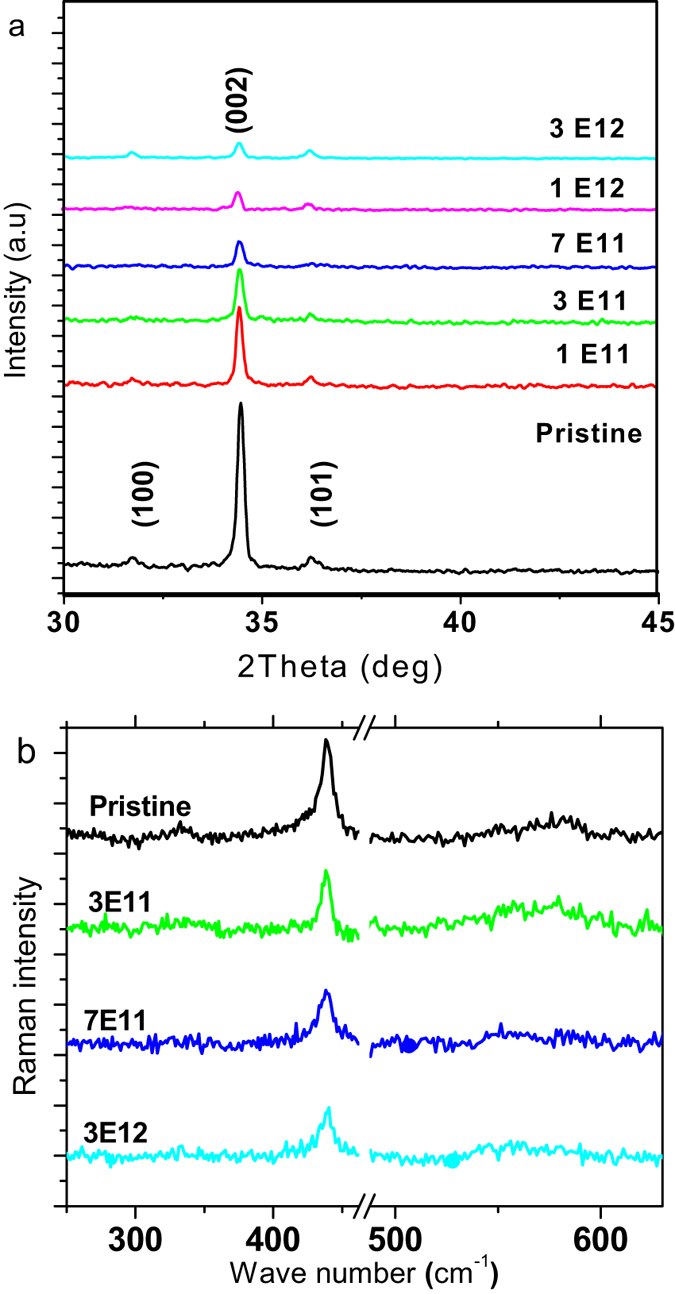
(**a**) X-ray diffraction spectra of the pristine and irradiated ZnO NR arrays at different fluences of 1E11, 3E11, 7E11, 1E12, 3E12 ions cm^−2^ with 150MeV Ag ions (**b**) Raman spectra of pristine and irradiated ZnO NR arrays, irradiated at the fluences of 3E11, 7E11 and 3E12.

Figure 4(a) shows the XRD patterns of ZnO NR arrays before and after irradiation at different fluences. The XRD spectrum of the pristine sample (see Fig. 4a) reveals that as-deposited ZnO NRs exhibit the presence of (002) single crystalline wurtzite hexagonal structure and there are no traces of any other secondary peaks corresponding to impurities. The variation of peak intensity of the most dominant (002) peak with respect to irradiation fluences was monitored. Under the influence of ion irradiation, there is detectable decrease in crystallinity of ZnO NRs induced due to disordering. On increasing the ion fluence, the ZnO NRs gradually undergo amorphization with increasing fluence.

Raman spectroscopy (Fig. 4b) is used to gain further insight into the nature of the defects present in the samples. Non-irradiated ZnO NR arrays exhibits the E_high_2 and a second order phonon of 2E_low_2, modes at 432 and 330 cm^−1^ respectively^29^. The significant features of the results presented in Fig. 4b are the drastic change in the intensity and broadening of the E2 peaks in the films subjected to ion irradiation. The silent Raman modes^30^ 552 cm^−1^ (B_high_1) are attributed to disorder-activated Raman scattering processes associated with the breakdown of the translational crystal symmetry that has been induced by the presence of defects. These defects state creation may be due to the induction of disorder on the ZnO NR arrays by ion irradiation. The irradiated films show that the intensity of the E_high_2 mode decreases drastically upon irradiation at 3E12 ions cm^−2^. The E_high_2 and E_low_2 modes are associated with the O and Zn sublattices^31^ respectively. Thus shifts in the intensity of the E_high_2 and E_low_2 modes would be consistent with large shifts in the concentrations of O and Zn vacancies and interstitials. Previously, it has been reported that the A_1_(LO) mode is affected by the presence of defects^32^. Hence, Raman studies confirm that the SHI irradiation treatment introduces disorder into the nanostructures, which certainly includes large concentrations of intrinsic point defects associated with the O and Zn sublattices.

### Optical studies

**Figure 5:**
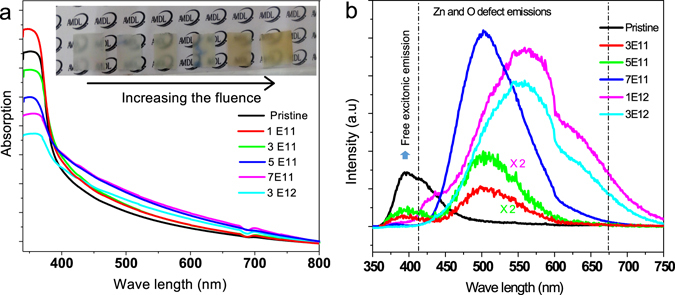
(**a**) Optical absorption spectra denoting band edge shift towards larger wavelength with increasing ion fluences. The photograph of the ZnO NR arrays exhibits changes in colour corresponding to increase in ion fluences (**b**) PL spectra of ZnO nanorods irradiated under different ion fluences. The PL emission defect intensity enhanced with ionic fluence, suggesting the enhancement of the oxygen defects in ZnO NRs. There is an appearance of emission peak at yellowish region above the ion fluence of 7E11 ions/cm^2^ indicating the creation of oxygen interstitials.

Figure 5a shows the absorption spectra of pristine and irradiated ZnO NR arrays. It can be observed from the spectra that the band edge is shifted towards larger wavelength on increasing the ion fluences. The decrease in the sharp band edge absorption indicates the formation of defect states (increased disorder states) in nanostructures. Figure 5b shows the photoluminescence spectra obtained from the Ag irradiated samples under different fluences of 3E11, 5E11, 7E11, 1E12 and 3E12 ions/cm^2^. PL results show typical two emissions of narrow violet (380 nm) and broad green-yellow bands (around 540 nm) (Fig. 5b). The broad emission bands revealed in the visible region in pristine ZnO sample is due to the superposition of green emissions. ZnO commonly exhibits luminescence in the visible spectral region due to different intrinsic or extrinsic defects^33^. According to the synthesis process as well as the assignments of defects in the literature discussed above, the defects formed in this system are likely to be due to oxygen vacancies and oxygen interstitials corresponding to the green and yellow bands in PL, respectively^34–36^. These two different (vacancies and interstitials) oxygen defects are competing with each other, presenting in the competition of green and yellow bands in PL. As seen in Fig. 5b, the PL emission defect intensity enhanced with ionic fluence, suggesting the enhancement of the oxygen defects in ZnO NRs. After the Ag ion fluence of 7E11 ions/cm^2^, the appearance of emission peak at yellowish region reveals the creation of oxygen interstitials caused at higher fluences. It can be concluded that abundant surface oxygen vacancies or defects exist in ZnO NRs, which depends on the ion irradiation fluences.

### Electronic Structures of ion irradiated ZnO NRs

**Figure 6:**
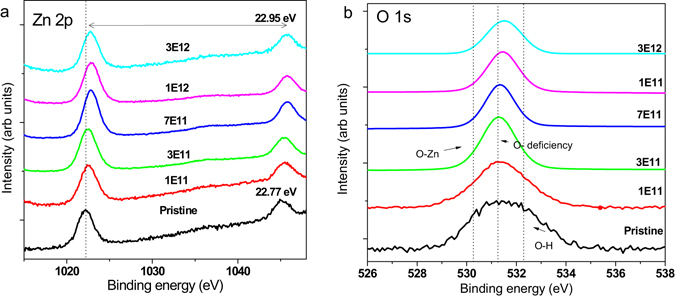
XPS analysis of Ag irradiated ZnO NR arrays in different fluences indicating the presence of structural deformations: (**a**) Zn 2p, and (**b**) O 1s spectra.

Figure 6 shows the XPS spectra of Ag ion irradiated ZnO NR arrays. Figure 6a shows the XPS spectra in the Zn 2p region. The spin orbit components (Zn-2p_3/2_ and 2p_1/2_) are observed at approximately 1021.5 and 1044.5 eV, and the splitting of the 2p doublet is around 23 eV indicating a normal state of Zn^2+^ in the ZnO NRs. Here, interestingly, on irradiation the 2p peak of ZnO was found to shift significantly to the higher binding energy compared to the standard value and pristine ZnO. The observed XPS peak shift can be explained, by change of electronic structure in nanocrystals, resulting in increase of binding energies^37^. From the previous investigations, it is clear that the Auger parameter is a useful tool to determine the stoichiometry and obtained phase in the crystal structure.

The main peak of the Zn (LMM) Auger series occurs around kinetic energy of 988 ± 0.5 eV. From the kinetic energy of Zn (LMM) and binding energy of Zn 2p_3/2_, we calculated the modified Auger parameter α′ (α′ = BE(Zn 2p_3/2_)+KE(Zn LMM), where BE and KE are binding energy of the core level 2p_3/2_ and a kinetic energy of the Auger transition involving electrons from L3, M4, and M5 core levels). For all samples, our calculations lead to a value around 2010.8 ± 0.7 eV, corresponding to ZnO (wurtzite) (Supplementary Table S1)^38^. On comparing the modified Auger parameter (a0) of the ion irradiated ZnO with the theoretical values, the pristine ZnO NRs matches almost perfectly when compared with the rest^38, 39^. However, for the ion irradiated ZnO NR arrays, a0 is significantly high and evidences different chemical states of Zn in these ion irradiated arrays. With respect to the XPS spectrum of O 1s in Fig. 6(b), the O1s signal was well shifted by the influence of ion irradiation from 529 eV to 533 eV. The peaks appeared at 529.9, 531.1, and 532.3 eV ascribed to Zn-O (lattice O), Oxygen deficiency or O vacancies, and Zn–OH, species, respectively. For pristine samples, there existed oxygen signals which were attributed to lattice O and oxygen related defect. But on ion irradiation, a notable shift from 530.7 eV (lattice oxygen vibration) to O-deficiency or vacancy at <531.2 eV is caused due to the oxygen defect and Zn-OH bond vibrations.

At the fluence of 7E11 ions/cm^2^, the ZnO NR arrays have strong oxygen defect ratio at 531.2 eV and 532.5 eV which are related to the oxygen signals attributed to O-deficiency and Zn-OH than the Zn-O lattice vibration. It is worth saying that ratio of oxygen vacancies and O-H increases with the ion fluences. This can be seen from the deconvoluted O1s peak showing notable shift from Zn–O bonding at 530.2 eV (lattice oxygen vibration) to Zn–O bonding at <532.5 eV (oxygen defect and Zn-OH bond vibrations) (see Supplementary Fig. S2). The influence on presence of higher O-H ratio over the ZnO NR surface may reduce the wettability at higher fluence.

### Durability and photoinduced wettability of Ag irradiated ZnO NRs

**Figure 7:**
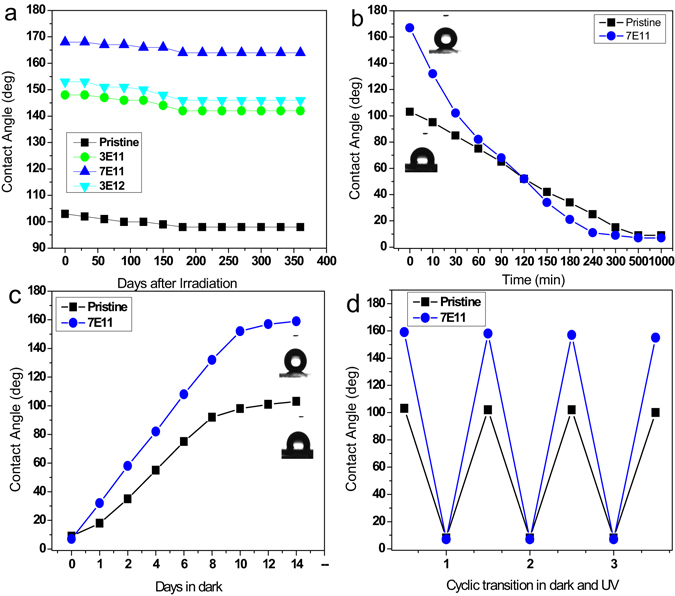
(**a**) Durability of the NR arrays for a period of one year. (**b**) Photoinduced water wettability transition from hydrophobic to hydrophilic in the ZnO NR arrays under UV illumination (**c**) water contact angle recovery in the dark-stored ZnO sample; Complete recovery to the initial state of superhydrophobicity is observed and (**d**) cyclic transition of water wettability with UV light illumination and dark storage. The pristine and irradiated ZnO NR arrays were subjected to UV light exposure and was stored in dark for a week before placing under UV illumination again.

Durability results reveal that the irradiated ZnO NR arrays show stable superhydrophobic behavior for nearly one year (Fig. 7a). We have also investigated water wetting behavior of ZnO NRs by studying the ultraviolet (UV) light induced wettability transition on pristine and irradiated ZnO NR arrays. Figure 7b shows the UV light induced wettability transition measurements on pristine and ion irradiated ZnO NRs. Under the UV light illumination, the oxygen defected surfaces are more favorable for the dissociative adsorption of the water molecules on the surface and these defective sites are more favourable for the hydroxyl groups (−OH adsorption than oxygen adsorption). It prompts the water adsorption and increases superhydrophilic surface behavior. The photogeneration of hydrophilic surfaces were dependent on the duration of UV light illumination as shown in Fig. 7b. On UV light illumination for about 300 min, the contact angle decreases from 168° ± 4° to 11° ± 2°. When these hydrophilic samples were stored in the dark for 14 days under ambient atmospheric conditions, the surfaces gradually reverted to superhydrophobic (Fig. 7c). The pristine and ion irradiated ZnO NR arrays display a reversible cyclic wetting transition from hydrophobic to hydrophilic and vice versa with UV light exposure and dark storage for a week as shown in Fig. 7d.

## Discussion

**Figure 8:**
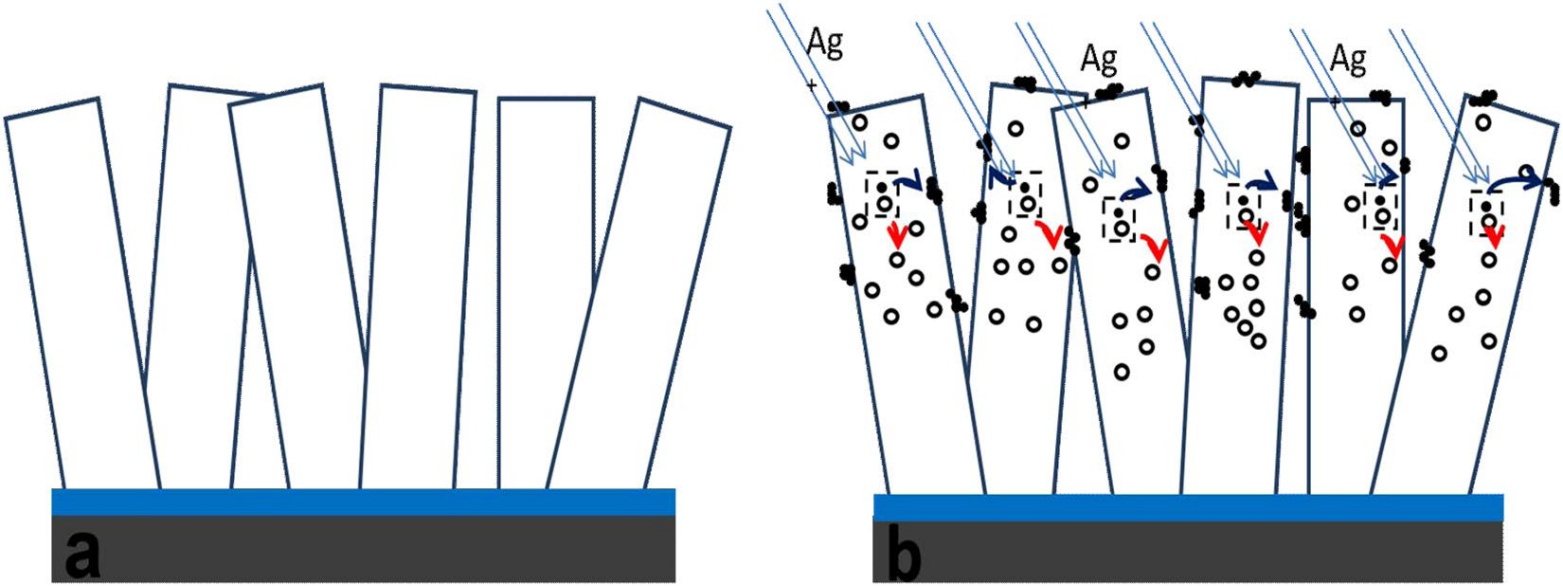
Schematic diagrams which show the generation, migration and accumulation of interstitials and vacancies in ZnO NRs (**a**) before and (**b**) after 150 MeV Ag^11^^+^ ion beam irradiation.

Creating the surface defect states through ion irradiation may play a dominant role in changing the wettability. The oxidized and oxygen deficiency surfaces effectively contribute to the surface energy. Oxidized surfaces exhibit high surface energy with the water molecules which can indeed make the surface hydrophilic^40^. Polarity of the surface through the oxygen based functional groups can control its wettability. Ion irradiation induced oxygen deficiency on the ZnO surface (oxygen vacancies), results in superhydrophobic surfaces. In contrast, higher ion fluences induce O interstitial formation or −OH sites on the ZnO surface, which reduces the CA. Upon comparison of the O vacancy defects with the water wetting behavior on the surfaces of ZnO NRs, it is found that the presence of surface O-vacancies in ZnO NRs provides extreme superhydrophobic behavior, whereas the O interstitial defect on ZnO NR surface renders nearly superhydrophobic nature. On irradiating the ZnO surfaces by SHI, it is expected that the damaging Ag^11^^+^ ions produce the Frenkel pairs, where the vacancies are accumulated in the crystal and the interstitial defects quickly move to the surface. Therefore, the interstitials may simply move to the surface and get agglomerated there. The schematic diagram in Fig. 8 illustrates the process of accumulation of vacancies and surface agglomeration of interstitials. Formation of these defected surfaces on ZnO nanostructures could alter the surface energy. Evolutions of change in these surficial properties are explained by the existing model of surface diffusion by scaling theory^41^.

The BH theory and low energy bombardment reveals the same phenomena for the surface evolution. But in present case the underlying mechanism of surface modification is believed to be due to the SHI bombardment. In our observation till now there are no theoretical or any experimental documentation available in literature for the modification of surface energy in ZnO NRs through ion bombardment, although there has been a report on creation of self-affine structures on ZnO thin films as a result of SHI irradiation^17^. Heavy ion irradiation, dominantly due to the electronic energy loss results in the electronic excitation and ionization of target by the inelastic collisions. There have been evidences of change in areal concentration of Zn and O under SHI irradiation with higher loss of O than Zn^17^. This result in the production of vacancies and interstitials on the ion beam irradiated ZnO surface and might facilitate the reduction of surface energy when compared to the pristine ZnO NR arrays. Collision effect through the ion irradiation clearly states that the amorphization of ZnO NRs increases with the fluence. The black patches are formed due to the SHIs which causes structural damages in the form of amorphous sites. When SHI passes through the material, it deposits large amount of energy into the material. According to the thermal spike model, the energy is deposited by the projectile ions in the electronic subsystem of the target. This energy is shared among the electrons by electron-electron coupling and transferred subsequently to the lattice atoms via electron-lattice interactions, leading to a large increase in the temperature along and in the vicinity of the ion path^42^. Pressure waves develop due to the temperature spike and cause strain in the nano structure. The diffusion of atoms within this zone is responsible for creating the defect enhanced surface of the nanostructures. As ion fluence increases, the fragmentation of these structures occurs due to the internal strain generated by ion beam, which results in increased amorphization of the nanostructures.

Ion bombardment of the ZnO NR arrays with a fluence of 7E11 ions/cm^2^ exhibited higher CA of 168° and on further increasing the fluence, the CA reduced. It may be due to the uncontrollable creation of interstitial defects and vacancy sites which favours uncontrollable inelastic collision (ionizing the target) that damages the ZnO surface. In our case, no model currently exists to give an exact possible explanation. Electronic sputtering in the ZnO nanoarrays gives clear evidence for the fact that the ions impart enough energy to atoms to make them mobile. The atoms which have energy more than the surface binding energy will escape the surface^43^. If the energy of the atoms is smaller than the binding energy, then these contribute to the surface diffusion.

The electronic excitations induced by SHI irradiations modify ZnO NR surfaces to superhydrophobic and superhydrophilic nature through the diffusion of self-affined defect states on ZnO NRs and their stability of dewetting properties were investigated. The result from the XRD, Raman and FESEM reveal that ion beam irradiation affects the surface properties of ZnO NR arrays which with further increase of irradiation lead to homogenization of defects. The irradiated energy contributes to the changes in the surficial properties of the NR arrays. Irradiated ZnO NRs are dominated by an almost uniformly distributed nanoscale self-affine oxygen related defect states in the NR structure. Formation of these self- defected surfaces on ZnO nanostructures occur due to the change in surface energy induced by energy deposition by SHIs in the near surface region.

## Methods

### Growth of ZnO NR arrays

ZnO NR arrays were synthesised by solution growth method. Nucleating ZnO seed layers were first deposited on glass substrates using dip coating method^44^. Equal mole ratio of Zinc acetate and ethylamine were dissolved in methoxyethanol and heated at 60 °C for two hours. ZnO seed layer was deposited on the substrate by dip coating and was dried at 150 °C. Dip coating process was repeated three to four times and the substrate was annealed at 350 °C. The ZnO seed layer coated substrate was immersed into a solution of equal mole of Zinc nitrate hexahydrate and Hexamine (HMTA) (C_6_H_12_N_4_) which were used as the precursors^45^. After immersing the substrate by facing top down, growth solution was maintained at 97 °C for 4 hours. After the growth time of 5h, the ZnO NR coated glass substrates were taken out and washed with DI water to remove the unwanted residual zinc salt impurities and dried in a stream of N_2_ and annealed at 250 °C for 2 hours.

### Characterization

The morphological and structural characterizations of as- synthesized samples were performed by field emission scanning electron microscopy (Carl Zeiss SUPRA 55) and X-ray diffraction spectrometer (Bruker Advanced D8) at IUAC, Delhi. Optical properties were studied using JASCO V620 spectrophotometer and Horiba Jobin Yvon Lab RAM (HR 800 Evolution) photoluminescence spectrometer using He−Cd laser of 325 nm wavelength. The vibrational characterization of ion irradiated ZnO was performed by Raman spectroscopy (Renishaw UK, Ar ion laser with 514.5 nm wavelength). The Fourier transform infrared (FTIR) spectroscopy measurements were done using Bruker Tensor 27 system through ATR mode. The chemical interactions were investigated by X-ray photoelectron spectroscopy using a SPECS monochromatic Al Kα X-ray source (hν 1486.6 eV) operating at 200 W, together with a PSP Vacuum Technology electron-energy analyzer operating with a constant pass energy of 10 eV. Calibration of the spectrometer was performed using a polycrystalline silver foil, cleaned in-vacuum. The Ag 3d5/2 photoelectron line had a binding energy of 368.3 eV and a FWHM of 0.6 eV. The wettability (static and rolling) of a water droplet on different ZnO NR array samples were characterized using an optical contact angle (CA) setup. A water droplet of 5 μL was used for each study, and the contact angle measurements were performed at five different positions for each sample. For inducing the superhydrophilicity on ZnO NR surface, UV light illumination was carried out by using a 9 W Phillips UV B lamp with the wavelength of 365 nm. The distance between the sample and UV lamp during illumination was 6 cm, and the measurements were done at room temperature.

### Ion Irradiation of ZnO NR arrays

ZnO NR arrays have been irradiated with 150 MeV Ag^11+^ ion beam with fluences of 1 × 10^11^, 3 × 10^11^, 5 × 10^11^, 7 × 10^11^, 1 × 10^12^ and 3 × 10^12^ ions cm^−2^ (hereafter will be represented as 1E11, 3E11, 5E11, 7E11, 1E12, 3E12 ions cm^−2^ respectively) at low temperature (80 K) using 15UD Pelletron tandem accelerator at Inter-University Accelerator Centre (IUAC), New Delhi. It was estimated from SRIM simulations that the electronic energy loss (S_e_) of Ag in ZnO is 17.6 keV/nm, while the nuclear energy loss (S_n_) is 0.067 keV/nm^46^. The samples were mounted on the sample holder, made of copper, in the high vacuum irradiation chamber of Materials Science Beamline. In order to achieve homogeneous irradiation, the ion beam was carefully raster scanned over an area of 1 × 1 cm^2^. Since the range (~15 µm) of the ions was greater than the film thickness, no ions were implanted into the ZnO film and modification was expected only due to the defects produced by ions passing through these ZnO NR arrays.

### Data availability

All data generated or analysed during this study are included in this published article (and its Supplementary Information files).

## Electronic supplementary material

Supplementary information 
