# Supplementary material for: Robust water repellent ZnO nanorod array by Swift Heavy Ion Irradiation: Effect of Electronic Excitation Induced Local Chemical State Modification
Source: Sci Rep. 2017 Jun 12;7:3251. doi: 10.1038/s41598-017-03313-8 (PMC5468313; doi:10.1038/s41598-017-03313-8)
Supplement: Supplementary file 1 — Supplementary information [file 41598_2017_3313_MOESM1_ESM.docx]

**Robust water repellent ZnO nanorod array by Swift Heavy Ion Irradiation: Effect of Electronic Excitation Induced Local**

**Chemical State Modification**

Kugalur Shanmugam Ranjith^1^, Lalitha Raveendran Nivedita^1^, Kandasami Asokan^2^, Satheesh Krishnamurty^3^, Ramanathaswamy Pandian^4^, Mohammed Kamruddin^4^, Devesh Kumar Avasthi^2,5^, Ramasamy Thangavelu Rajendra Kumar^1,6*^

*^1^Advanced Materials and Devices Laboratory, Department of Physics, Bharathiar University, Coimbatore 641046, India*

*^2^Materials Science Division, Inter University Accelerator Centre, New Delhi-110 067, India.*

*^3^School of Engineering and Innovation, The Open University MK 76AA, UK.*

*^4^Surface and Nanoscience Division, Materials Science Group, Indra Gandhi Centre for Atomic Research (IGCAR), Kalpakkam 603102, India.*

*^5^Amity Institute of Nanotechnology, Noida 201313, India*

*^6^Department of Nanoscience and Technology, Bharathiar University, Coimbatore 641046, India*

**Email:[rtrkumar@buc.edu.in](mailto:rtrkumar@buc.edu.in)*

Figure S1. Survey spectrum of ZnO NR arrays after Ag irradiation under different fluences

f

e

d

c

b

a

Figure S2. Gaussian multi fit function over the O 2s XPS spectra of Ag irradiated ZnO NR arrays in different fluences indicating the presence of structural deformations (a) pristine, (b) 3E11 (c) 5E11, (d) 7E11, (e) 1E11, (e) 3E12 ions cm^-2^.

Table S1. XPS results presenting the position and the relative contribution of the Zn–OH and Zn–O bonding for films irradiated at different fluences

| Sample | Peak | Peak position  BE (±0.1 eV) | Shift evolved (eV) | Zn LMM  (±0.1 eV) | Modified Auger  Parameter α (eV) | Peak | BE (±0.1eV) | Shift evolved (eV) | FWHM (±0.2 eV) of O-Zn | Defect Relation contribution % |
| --- | --- | --- | --- | --- | --- | --- | --- | --- | --- | --- |
| Pristine | Zn2p | 1022.17 | Nil | 988.04 | 2010.21 | O1s (Zn-O)  O1s (O deficiency)  O1s (Zn-OH) | 530.18  531.31  532.53 | Nil | 2.994 | 52  28  20 |
| 3E11 g) low temp | Zn2p | 1022.43 | 0.26 | 988.61 | 2011.04 | O1s (Zn-O)  O1s (O deficiency)  O1s (Zn-OH) | 530.24  531.50  532.72 | 0.25 | 2.112 | 32  52  16 |
| 5E11 (150 MeV Ag) low temp | Zn2p | 1022.51 | 0.34 | 988.67 | 2011.22 | O1s (Zn-O)  O1s (O deficiency)  O1s (Zn-OH) | 530.51  531.82  532.82 | 0.31 | 2.199 | 18  60  22 |
| 7E11 (150 MeV Ag) low temp | Zn2p | 1022.81 | 0.64 | 988.69 | 2011.50 | O1s (Zn-O)  O1s (O deficiency)  O1s (Zn-OH) | 530.60  531.52  532.58 | 0.34 | 2.101 | 15  56  29 |
| 1E12 (150 MeV Ag)low temp | Zn2p | 1022.94 | 0.77 | 988.59 | 2011.53 | O1s (Zn-O)  O1s (O deficiency)  O1s (Zn-OH) | 530.24  531.38  532.47 | 0.42 | 2.165 | 22  47  31 |
| 3E12 (150 MeV Ag) low temp | Zn2p | 1022.78 | 0.61 | 988.42 | 2011.20 | O1s (Zn-O)  O1s (O deficiency)  O1s (Zn-OH) | 530.22  531.33  532.64 | 0.46 | 2.296 | 17  39  44 |
